# Zonisamide ameliorates progression of cervical spondylotic myelopathy in a rat model

**DOI:** 10.1038/s41598-020-70068-0

**Published:** 2020-08-04

**Authors:** Shunsuke Kanbara, Bisei Ohkawara, Hiroaki Nakashima, Kyotaro Ohta, Hiroyuki Koshimizu, Taro Inoue, Hiroyuki Tomita, Mikako Ito, Akio Masuda, Naoki Ishiguro, Shiro Imagama, Kinji Ohno

**Affiliations:** 1 Department of Orthopedic Surgery, Nagoya University Graduate School of Medicine, 65 Tsurumai, Showa-ku, Nagoya, 466-8550 Japan; 2 Division of Neurogenetics, Center for Neurological Diseases and Cancer, Nagoya University Graduate School of Medicine, 65 Tsurumai, Showa-ku, Nagoya, 466-8550 Japan

**Keywords:** Drug regulation, Spinal cord diseases, Spinal cord injury

## Abstract

Cervical spondylotic myelopathy (CSM) is caused by chronic compression of the spinal cord and is the most common cause of myelopathy in adults. No drug is currently available to mitigate CSM. Herein, we made a rat model of CSM by epidurally implanting an expanding water-absorbent polymer underneath the laminae compress the spinal cord. The CSM rats exhibited progressive motor impairments recapitulating human CSM. CSM rats had loss of spinal motor neurons, and increased lipid peroxidation in the spinal cord. Zonisamide (ZNS) is clinically used for epilepsy and Parkinson's disease. We previously reported that ZNS protected primary spinal motor neurons against oxidative stress. We thus examined the effects of ZNS on our rat CSM model. CSM rats with daily intragastric administration of 0.5% methylcellulose (*n* = 11) and ZNS (30 mg/kg/day) in 0.5% methylcellulose (*n* = 11). Oral administration of ZNS ameliorated the progression of motor impairments, spared the number of spinal motor neurons, and preserved myelination of the pyramidal tracts. In addition, ZNS increased gene expressions of cystine/glutamate exchange transporter (xCT) and metallothionein 2A in the spinal cord in CSM rats, and also in the primary astrocytes. ZNS increased the glutathione (GSH) level in the spinal motor neurons of CSM rats. ZNS potentially ameliorates loss of the spinal motor neurons and demyelination of the pyramidal tracts in patients with CSM.

## Introduction

Cervical spondylotic myelopathy (CSM) is caused by chronic progressive degeneration of the cervical spine with a prevalence of 4.04 per 100,000 persons-years^1,2^. While more than 50% of middle-aged adults have radiographic evidence of cervical alteration, 10% of them have clinically significant radiculopathy or myelopathy^3–7^. CSM occurs when the spinal cord becomes compressed by bone spurs, herniated spinal disc, and ligamentous hypertrophy, and so on^8,9^. Although surgical intervention can attenuate the progression of CSM, most patients are still left with significant neurological impairments^10–14^.

The histological features of CSM include gliosis, loss of anterior horn (AH) cells, and cystic necrosis resulting from the chronic and mechanical compression^15–17^. However, the exact cellular mechanisms for insidious and progressive deterioration remain not to be fully elucidated^9^. Recent studies show that chronic compression of the spinal cord sequentially causes disruption of the blood-spinal cord barrier, ischemia, and neuroinflammation^5,16^. CSM is characterized by loss of spinal motor neurons, degeneration of axons constituting the corticospinal tracts, demyelination of oligodendrocytes, astrocytic scarring, and cavitation^4,5,18^. Neuronal and oligodendrocytic apoptosis is observed in human CSM and in the Tiptoe Walking Yoshimura (twy/twy) hyperostotic mice^15–17^. Multicellular apoptosis in CSM is accounted for by glutamatergic toxicity, free radicals (oxygen radicals and lipid peroxidation), and cationic cell injury^16^.

Zonisamide (ZNS) is an anti-epileptic agent widely used as an adjunctive therapy for partial seizures in adults and an anti-Parkinsonian agent particularly for resting tremor^19^. We previously reported that ZNS enhanced neurite elongation of primary spinal motor neurons in mice, and facilitated nerve regeneration of an autograft of the sciatic nerve in mice^20,21^. We also reported that ZNS protected primary spinal motor neurons against oxidative stress^20,21^. In a mouse model of Parkinson’s disease, ZNS counteracts neurodegeneration partly by increasing expressions of cystine/glutamate exchange transporter (xCT) in astrocytes and glutathione (GSH) in the dopaminergic neurons in the striatum^22^. xCT in astrocytes takes up cystine, which is converted into two cysteines^23,24^. Neurons then take up astrocyte-derived cysteine, and synthesize GSH. Thus, the synthesis of GSH in neurons is dependent on the expression of xCT in astrocytes^22^. In addition to xCT, ZNS also induces the expression of metallothionein 2A, which captures radical residues as an anti-oxidant, in response to oxidative stress, in astrocytes^22,25^. ZNS is thus likely to exert its neuroprotective effect as an anti-oxidant.

In this study, we made a rat model of CSM at cervical (C) levels 5 and 6. The CSM rats showed progressive motor impairments, loss of spinal motor neurons, and increased lipid peroxidation represented by 4-hydroxynonenal (4-HNE). The effects of ZNS on nerve regeneration^20,21^ and on preventing degeneration of dopaminergic neurons^22,25^ prompted us to examine the effects of ZNS on a rat CSM model. We found that oral administration of ZNS delayed progression of motor impairments, rescued loss of spinal motor neurons, and spared myelination of the pyramidal tracts. ZNS also upregulated xCT and metallothionein 2A in the spinal cord, which is associated with increased GSH production in the motor neurons.

## Materials and methods

### Animal maintenance and group design

All rat studies were approved by the Animal Care and Use Committee of Nagoya University, and were performed in accordance with relevant guidelines. All rats were housed in environmentally adequate cages with less than 3 animals. The temperature was maintained at 22 °C and the room was kept at a 12/12-h light/dark cycle.

Adult female Wistar rats aged 12 weeks were purchased from Japan SLC, Inc. Two independent experiments were performed. The first experiment included two groups of sham-operated (*n* = 13) and CSM (*n* = 7) rats. The second experiment included CSM rats with daily intragastric administration of 0.5% methylcellulose (*n* = 11) and ZNS (30 mg/kg/day) in 0.5% methylcellulose (*n* = 11).

### Surgical implantation of the expandable material

We used an expandable material made of a water-absorbing polyurethane elastomer (Aquaprene Dx, Sanyo Chemical Industries)^26^. The polymer sheet reached a volume of 150% over 24 h when embedded under the skin of a rat (Supplemental Fig. S1A). The polymer was dissected in a rectangular shape with 0.80 mm (ventrodorsal) × 3 mm (mediolateral) × 5 mm (rostrocaudal) using a Mcilwain Tissue chopper (Campden Instruments LTD, England). The sizes of the implant were essentially taken from previous reports^27,28^ except that we increased the thickness from 0.70 mm to 0.80 mm. To avoid injury to the spinal cord, the edge of the polymer was slanted by removing 1 mm from the corner using a surgical knife (Supplemental Fig. S1B). We attached a surgical thread (3-0 Ethicon, Johnson & Johnson K. K.) and used it to guide the passage of the blade underneath the C5 and C6 laminae.

Anesthesia was induced by inhaling 2.5% isoflurane gas (Fujifilm Wako Pure Chemical) through a facial mask. We implanted the blade essentially following a protocol by Kim et al.^28^. Briefly, a 4.0-cm midline skin incision was made from C2 to the thoracic (T) level 2. The C4-7 laminae were identified by counting spinous processes from T2, and were carefully exposed. The ligamenta flava at C4-5, C5-6, and C6-7 were removed to expose the underlying dura. The blade was epidurally implanted underneath the C5 and C6 laminae using microsurgical techniques (Supplemental Fig. S1C–E), and multilayer tissue closure was performed. The sham-operated group had mock surgery without implantation.

### Oral administration of Zonisamide (ZNS)

Thirty mg of ZNS (Lot# 1422, Sumitomo Dainippon Pharma) was dissolved in 10 ml of 0.5% methylcellulose, and 30 mg/kg/day ZNS was intragastrically administered using a disposable sonde needle (flexible type, Fuchigami), as we previously described ^22^. ZNS( +) group had ZNS administered every day for 5 weeks after surgery, whereas ZNS(-) group were administered with 0.5% methylcellulose every day.

### Wire mesh walking test

The numbers of forepaw and hindpaw slips were counted using a 100 cm × 10 cm walkway with a 3.0 × 3.0-cm grid made of 1.6-mm-thick wires. Videos were recorded for each animal while completing the walk three times^9,29^. A slip was counted when one of paws fell below the plane of the grid, and the average number of slips during a 100-cm walk was calculated. The percentage of paw slips was calculated by dividing the total number of slips by the total number of steps^30^.

### Automated gait analysis (CatWalk)

Gait analysis was performed using the CatWalk system XT Ver. 9 (Noldus Information Technology, Wageningen, Netherlands) at 1 to 10 weeks after the surgical implantation, as described previously^31–34^. Briefly, the system was consisted of a horizontal glass plate with computer-operated video-recording equipment placed underneath the glass plate. The following criteria were used for walkway crossing: (1) the rat needed to cross the walkway without any interruption, and (2) a minimum of three complete crossings per animal were required. We evaluated the stride length and the duty cycle with the CatWalk system. The duty cycle indicates a ratio (%) of the time when each paw is in contact with the glass plate in a single step cycle. We also measured the print position, which is the distance between the placements of the ipsilateral fore- and hind-paws. Data of gait factors were obtained from the both side limbs and the values were calculated independently.

### Histopathology

CSM rats were histologically analyzed at 5–10 weeks after surgical implantation. Rats were transcardially perfused with 4% paraformaldehyde (PFA) in cold phosphate-buffered saline (PBS) at pH 7.4. The spine at C2-T2 was removed and fixed in 4% PFA and 10% sucrose overnight at 4 °C. Laminae and vertebral bodies were removed, and a 5-mm-thick segment of the spinal cord at C5-6 was isolated. The C5-6 segment was embedded in paraffin and sectioned into 9 μm-thick slices. The specimens were stained with hematoxylin/eosin (HE) and Luxol Fast Blue/Nissl (LFB/Nissl). Neurons in the anterior horn region were identified by the presence of large nuclei and the Nissl bodies in the cytoplasm. Number of the neurons was counted in the both sides of anterior horn region and evaluated independently. For frozen sections, a 5-mm-thick segment at C5-6 was similarly prepared as stated above, and was secondarily fixed with 4% PFA and 20% sucrose in PBS overnight at 4 °C. Subsequently, the C5-6 segment was sectioned into 20 μm-thick slices.

### Immunohistochemistry

Lipid peroxidation was evaluated by immunostaining for 4-HNE of paraffin sections of the spinal cord. The sections were blocked with 10% goat serum (Fujifilm Wako Pure Chemical, WDR2410) in PBS, and were incubated with an anti-4-HNE monoclonal antibody (1:40, JaICA, MHN-100P) at 4 °C overnight. The sections were then incubated with a biotinylated anti-mouse IgG antibody (1:100, Vector Laboratories, BA-2000) followed by incubation with an avidin–biotin-horseradish peroxidase complex (1:500, Vector Laboratories, the Vectastain ABC kit), and were visualized with the ImmPACT DAB Substrate (Vector Laboratories). The number of 4-HNE-positive cells were counted in the both side of anterior horn region and evaluated independently. The frozen sections were used for staining GSH, choline acetyltransferase (ChAT), and 4,6-diamidino-2-phenylindole (DAPI). After blocking with 5% horse serum (Invitrogen) and 0.3% Triton X-100 in 0.1 M PBS, the sections were air-dried at room temperature for 10 min, placed in cold acetone for 10 min, and dried again. The sections were then incubated overnight at 4 °C with the following primary antibody: mouse anti-GSH (1:1,000, Abcam, ab19534) or goat anti-ChAT (1:100, Millipore, AB144P). The sections were washed in PBS three times and incubated with biotinylated anti-goat IgG (1:100, Vector laboratories, BA-9500) for 2 h, washed in PBS three times, and incubated with Alexa 546-conjugated streptavidin (1:100, Invitrogen, S11225) or FITC-conjugated anti-mouse IgG (1:100, Vector Laboratories, FI-2000) for 30 min. The sections were mounted on cover slips with Mowiol mounting medium containing DAPI to counterstain the nuclei. Immunostaining was quantified by two blinded observers using the BZ-9000 microscope (Keyence, Woodcliff Lake, NJ). In 4-HNE-stained sections, a diameter of > 40 μm with clear cytoplasm was used as a hallmark of spinal motor neurons. The intensity of GSH signals co-localized with ChAT signals in the gray matter in CSM rats was quantified by two blinded observers using a confocal laser scanning microscope system (TiE-A1R, Nikon) and the Image-J software (NIH). The intensity of GSH signals co-localized with ChAT signals were counted in the both side of anterior horn region and evaluated independently.

### Total RNA extraction and quantitative RT-PCR

Sham-operated rats and CSM rats with or without ZNS at 10 weeks after surgery were transcardially perfused with PBS. The spinal cord at C5-6 was isolated as stated in the section for histopathology. Total RNA of the spinal cord was isolated using QIAzol (QIAGEN). First strand cDNA was synthesized with ReverTra Ace (Toyobo). cDNA was quantified in triplicates using the LightCycler Master Mixes (Roche) on LightCycler 480 (Roche). The mRNA levels were normalized for those of β-actin. Primer sequences are shown in Supplemental Table S1.

### Statistical analysis

**Figure 1:**
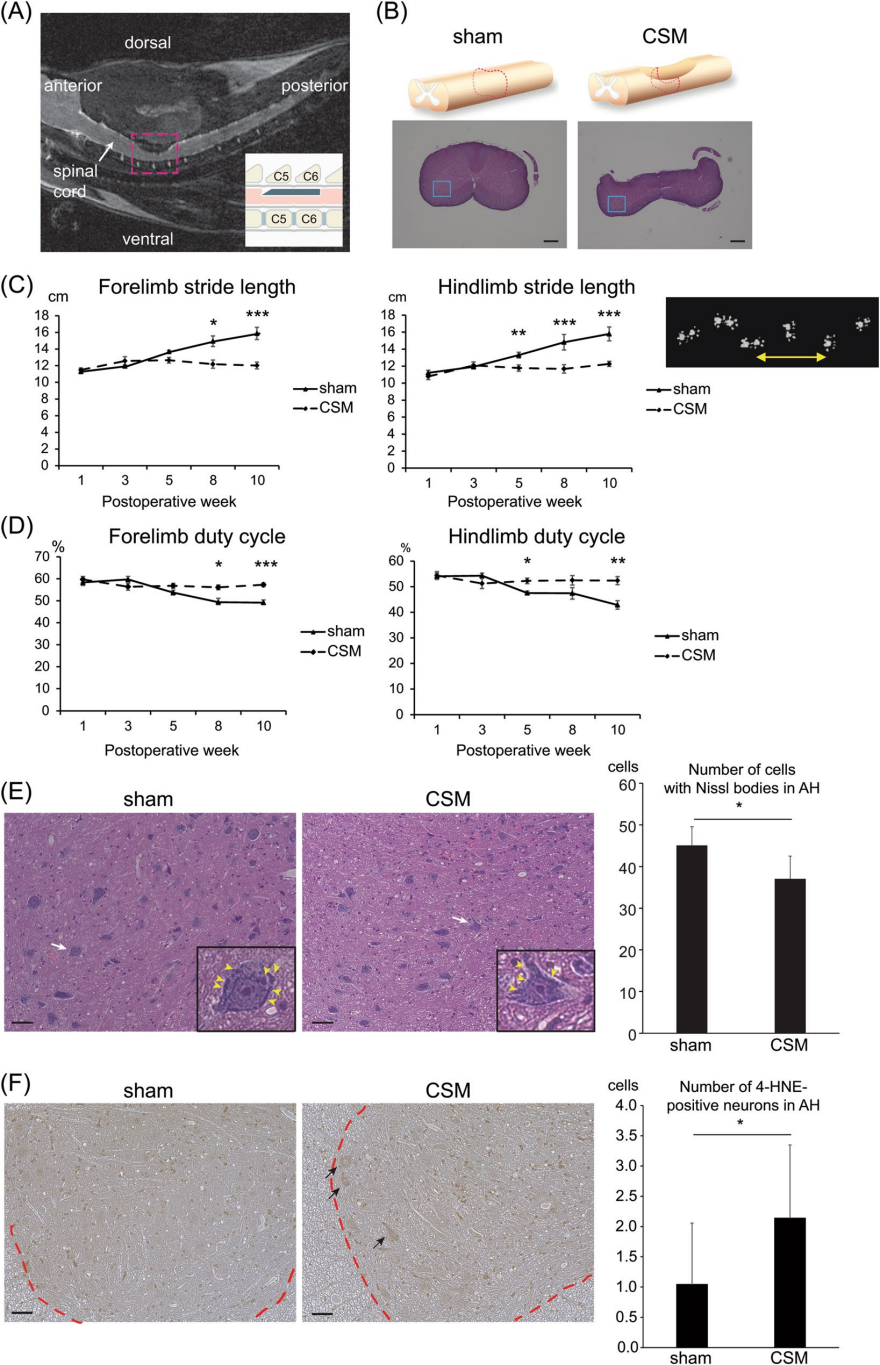
Motor deficits and histopathologies of a rat model of CSM. (**A**) A representative T2-weighted mid-sagittal MRI image of a CSM rat at 10 weeks after surgery shows dorsal–ventral cervical spinal cord compression at C5 and C6 levels while keeping laminae of C4, 5, 6, and 7 intact. The area in the red dotted square is schematically shown in the inset. (**B**) Upper panels schematically show the spinal cord around the C5-6 level. Hematoxylin and eosin (HE) staining of axial sections at the red broken lines at the C5-6 disc level are shown in the lower panels. Blue boxed areas are enlarged in (**E**). Bar = 600 μm. (**C**,**D**) Stride length (**C**) and duty cycle (**D**) of fore- and hind-limbs analyzed by the CatWalk system of sham-operated and CSM rats from 1 to 10 weeks postoperatively. (**C**) A representative stride length of the right forelimb is indicated by a double-headed arrow in the right panel. (**D**) Duty cycle is a ratio in time when a paw stays on the glass plate in a single step cycle. CSM rats started to exhibit gait disturbance from 5 weeks after surgery. Mean and SE are indicated (*n* = 6 and 4 for sham-operated and CSM rats, respectively). *p < 0.05, **p < 0.01, and ***p < 0.001 by two-way repeated measures ANOVA followed by Bonferroni correction. (**E**,**F**) HE staining at 5 weeks postoperatively (**E**) and 4-hydroxynonenal (4-HNE) staining at 10 weeks postoperatively (**F**) of an axial section of the spinal cord at the C5-6 disc level in sham-operated and CSM rats. (**E**) Images are enlargement of the blue boxed areas in (**B**). A representative neuron (white arrow) in the anterior horn (AH) region is enlarged in the inset. Purple-stained Nissl bodies are indicated by yellow arrowheads. The numbers of cells with Nissl bodies in the AH region of sham-operated and CSM rats are shown. Mean and SD are indicated (*n* = 3 rats in each group). *p < 0.05 by Mann–Whitney U-test. (**F**) Representative images of the spinal cord stained with 4-hydroxynonenal (4-HNE). Arrows point to 4-HNE-positive large cells (diameter > 40 μm), which were recognized as motor neurons, in the AH region. A border between the gray and the white matters is indicated by a red dotted line. Bar = 50 μm. Mean and SD are indicated (*n* = 3 rats in each group). *p < 0.05 by Mann–Whitney U-test.

**Figure 2:**
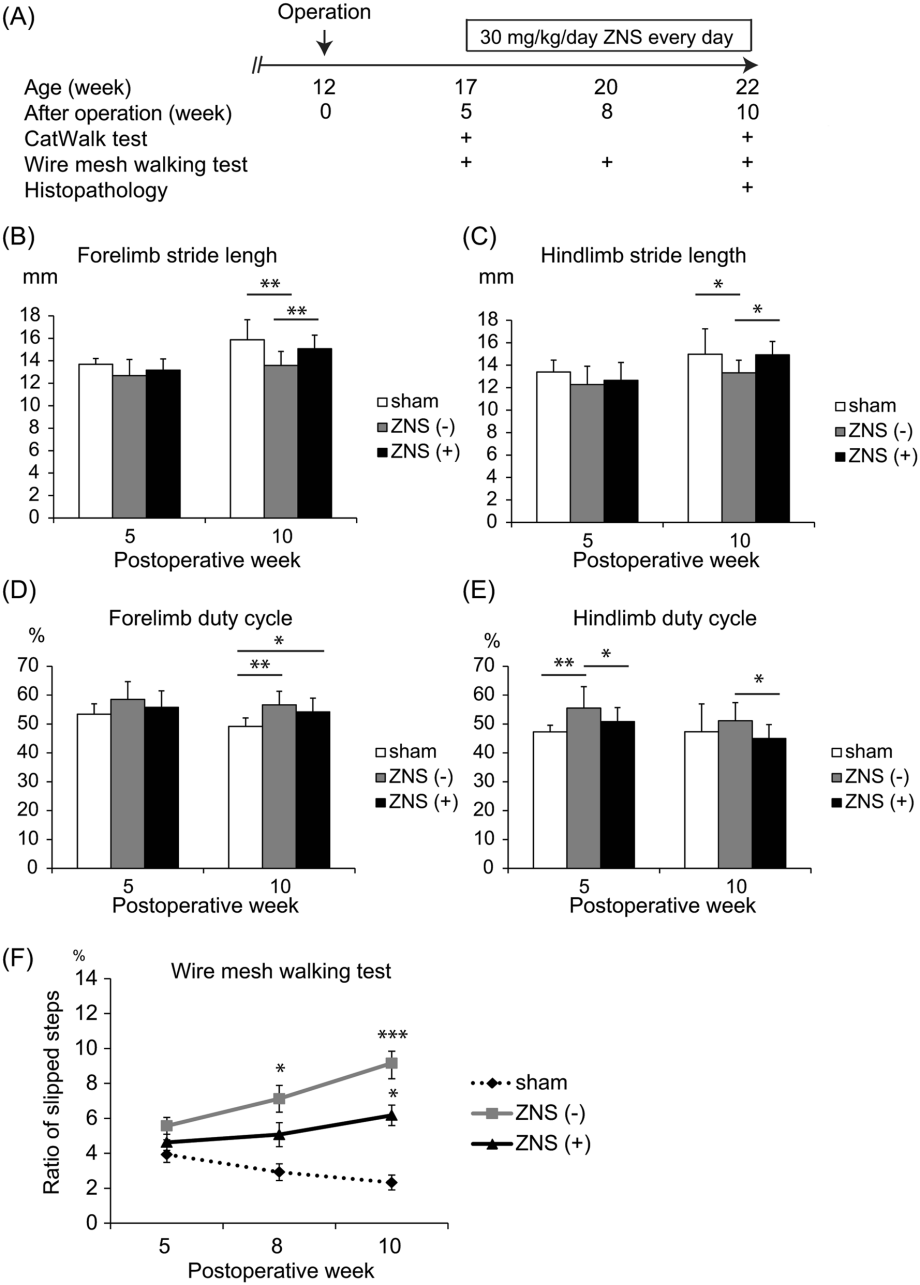
Temporal profiles of motor deficits of CSM rats with or without Zonisamide (ZNS) administration. (**A**) Experimental protocol of gait and histological analyses of CSM rats. Water-absorbing polyurethane blade was implanted at 12 weeks of age. Operated rats were divided into three groups: sham-operated [sham; *n* = 6 rats], daily intragastric administration of 30 mg/kg ZNS in 0.5% methylcellulose [ZNS( +); *n* = 11 rats] and 0.5% methylcellulose alone as a control [ZNS(-); *n* = 11 rats]. (**B–E**) Stride length (**B** and **C**) and the duty cycle (**D** and **E**) of fore- and hind-limbs were analyzed with CatWalk. At 10 weeks after surgery, ZNS increased the stride length of forelimbs (**B**) and hindlimbs (**C**), and reduced the duty cycle of hindlimbs (**E**). * *p* < 0.05 and ** *p* < 0.01 by one-way ANOVA followed by Tukey HSD. (**F**) Ratio of slipped steps on a ladder bar was analyzed by the wire mesh walking test. Mean and SD are indicated (*n* = 12 limbs in 6, 22 limbs in 11, and 22 limbs in 11 for sham-operated, ZNS(-) and ZNS( +) rats, respectively). * *p* < 0.05 and *** *p* < 0.001 by two-way repeated measured ANOVA followed by Bonferroni correction.

**Figure 3:**
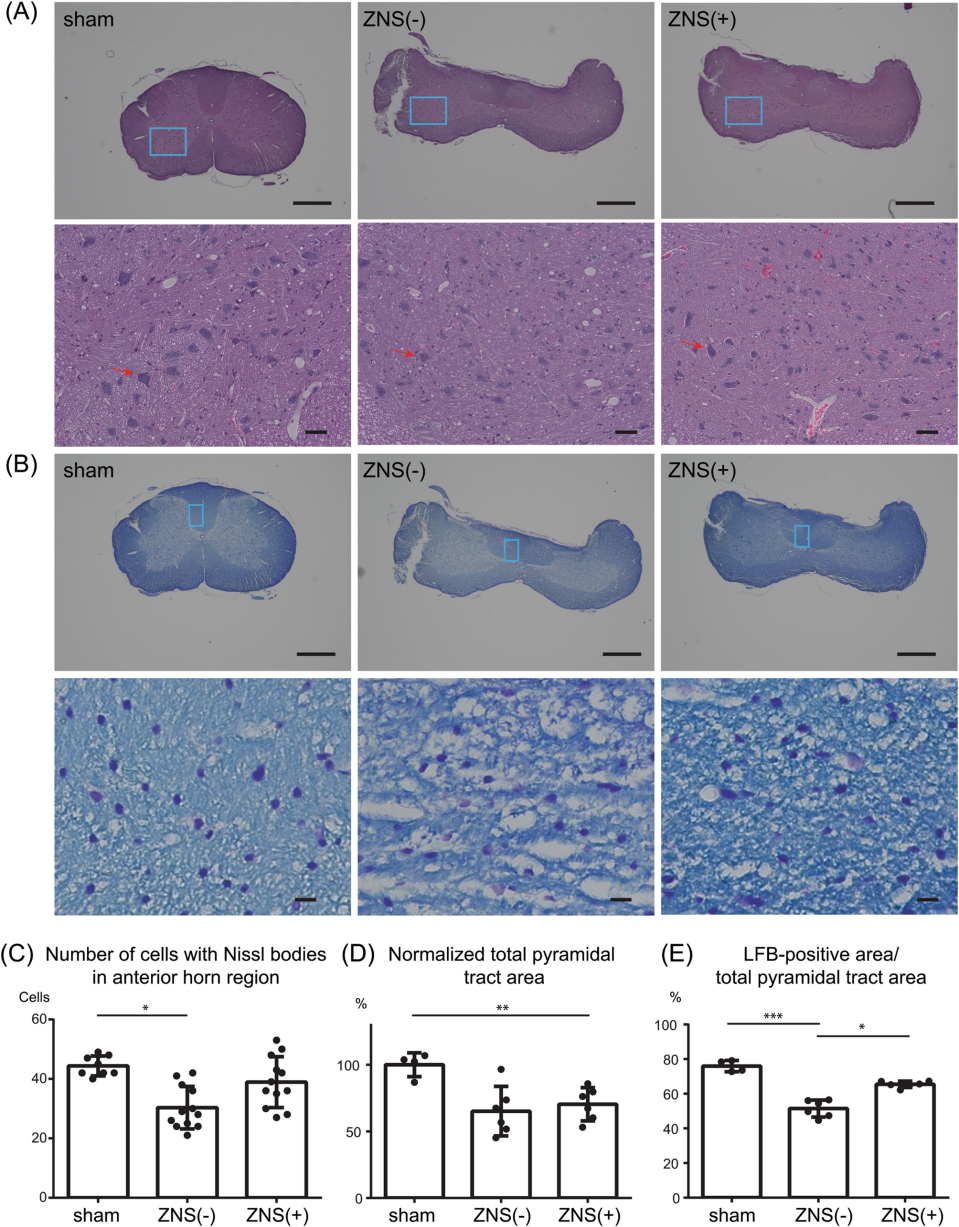
Effects of Zonisamide (ZNS) on the number of neurons in the anterior horn (AH) region and on demyelination of the pyramidal tracts. (**A–B**) Representative low (upper panels) and high (lower panels) magnification images of hematoxylin and eosin (HE) staining (**A**) and Luxol Fast Blue (LFB)/Nissl staining (**B**) of the spinal cord at the C5-6 disc level at 10 weeks after CSM operation. Blue squares in the AH region (**A**) and the pyramidal tract (**B**) in upper panels are magnified in lower panels. (**A**) A representative neuron in each panel is indicated by red arrow. Bars in upper panels = 600 μm. Bars in lower panels = 50 μm. (**C**) The number of cells with Nissl bodies in the AH region. (**D**) Total area of the pyramidal tracts was measured and normalized to that in sham-operated rat. The pyramidal tracts were compressed by a water-absorbing polyurethane blade in both ZNS(-) and ZNS( +) rats. (**E**) The area of LFB (light blue)-stained myelin was divided by the total area of pyramidal tracts in the spinal cord at the C5-6 disc level. (**C**,**D**,**E**) Mean and SD are indicated [*n* = 8, 12, and 12 anterior horns in 4 sham-operated, 6 ZNS(-), and 6 ZNS( +) rats, respectively]. * *p* < 0.05, ** *p* < 0.01, and *** *p* < 0.001 by Kruskal–Wallis test followed by Dunn-Bonferroni HSD.

**Figure 4:**
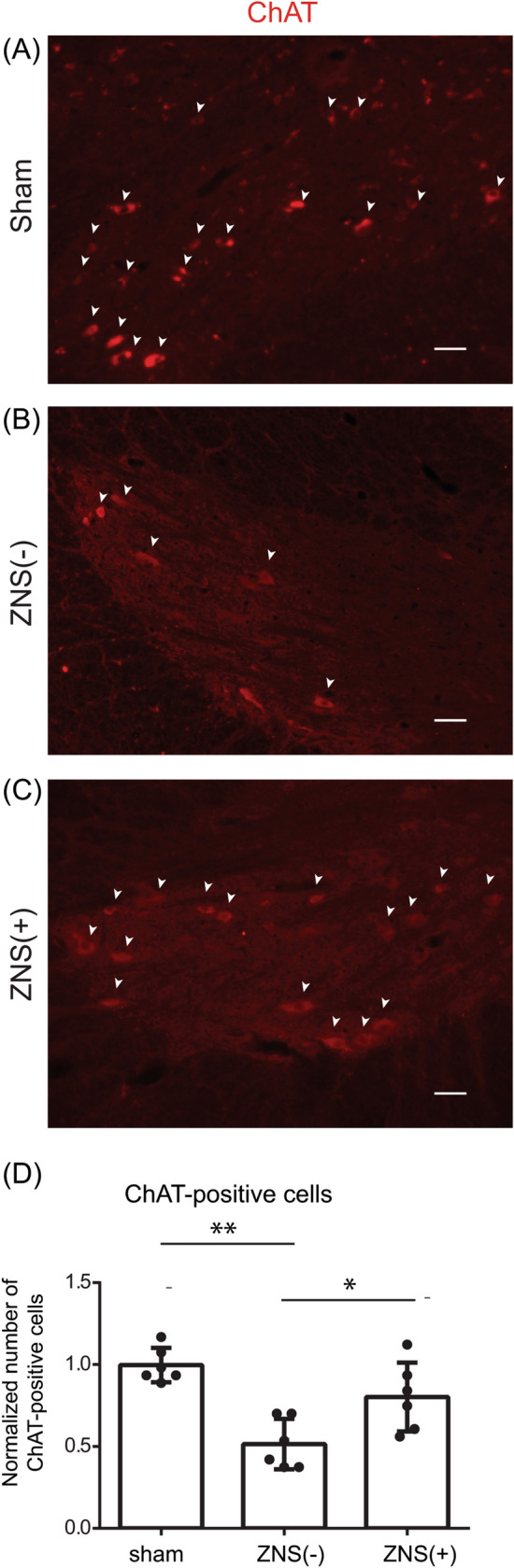
Effect of Zonisamide (ZNS) on the number of ChAT-positive motor neurons in the anterior horn (AH) region. (**A–C**) Representative confocal images of the anterior horn (AH) region in the spinal cord at the C5-6 disc level of sham-operated rat and CSM rat with [ZNS( +)] or without [ZNS(-)] ZNS administration. Sections were immunostained with an antibody against choline acetyltransferase (ChAT, red). ChAT-positive cells are indicated by white arrowheads. Bar = 50 μm. (**D**) The number of ChAT-positive cells in the AH region in sham-operated, ZNS(-) and ZNS( +) rats. Mean and SD are indicated (*n* = 6 anterior horns of 3 rats for each group). * *p* < 0.05, and ** *p* < 0.01 by Kruskal–Wallis test followed by Dunn-Bonferroni HSD.

**Figure 5:**
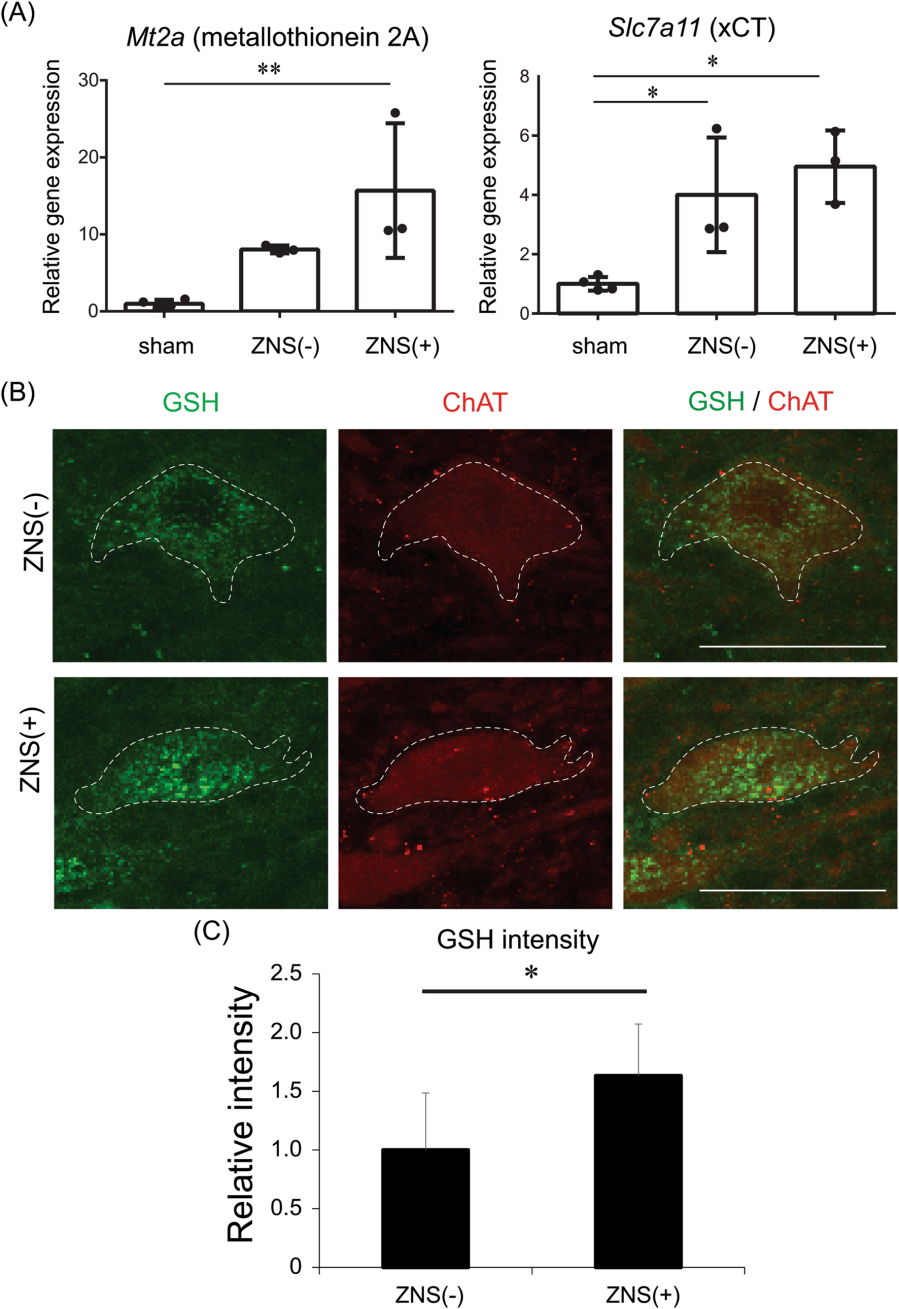
Effects of Zonisamide (ZNS) on mRNA expressions of metallothionein 2A and xCT in the spinal cord, and on immunostaining for glutathione (GSH) in spinal motor neurons in CSM rats. (**A**) Effect of ZNS on mRNA expressions of *Mt2a* encoding metallothionein 2A and *Slc7a11* encoding cystine/glutamate exchange transporter (xCT) in the spinal cord at the C5-6 levels in sham-operated and CSM rats with [ZNS( +)] or without [ZNS(-)] ZNS. Means and SD are indicated (*n* = 4, 3, and 3 for sham-operated, ZNS(-), and ZNS( +) rats, respectively). *p < 0.05 and **p < 0.01 by Kruskal–Wallis test followed by Dunn-Bonferroni HSD. (**B**) Representative confocal images of GSH (green)- and ChAT (red)-positive cells in the anterior horn (AH) region of the spinal cord at the C5-6 disc level in CSM rat. Note that spotty cytoplasmic staining for GSH is observed in ChAT-positive motor neurons outlined by a dotted line. Low-magnification images of these panels are indicated in Supplemental Fig. S3. Bar = 50 μm. (**C**) Relative intensity of GSH signals in ChAT-positive motor neurons in the AH region in CSM rats with [ZNS ( +)] or without [ZNS (-)] ZNS administration. Mean and SD are indicated (*n* = 3 rats in each group). * *p* < 0.05 by Mann–Whitney U-test.

Motor functions were all analyzed in a blinded manner. Statistical analysis was performed using SPSS ver. 21 (IBM Corp.). Figures 1CD and 2F were analyzed by two-way repeated measures ANOVA followed by Bonferroni correction. Figures 1E,F and 5C were analyzed by Mann–Whitney U test. Figure 2B–E were analyzed by one-way ANOVA followed by Tukey honestly significant difference (HSD) test, as Fig. 2B–E were each comprised of 10 or more samples. Figures 3C–E, 4D, 5A, and S2A-C were analyzed by Kruskal–Wallis test followed by Dunn-Bonferroni HSD test, as the sample sizes were less than 10 in each group. *P* values of 0.05 or less were considered to be statistically significant.

## Results

### CSM rats exhibit gait dysfunctions from 5 weeks after surgery

In patients with CSM, motor and sensory symptoms develop slowly and progress steadily for several years^35–37^. To recapitulate human CSM in an animal model, we epidurally implanted a blade-shaped water-absorbing polyurethane elastomer (Aquaprene Dx) ^26^ underneath the C5 and C6 laminae in a rat, while keeping the laminae uncut (Fig. 1A,B and Supplemental Fig. S1). The sham-operated rats had mock surgery without implantation.

We evaluated motor dysfunctions and histopathology of the spinal cords in our CSM rats (Supplemental Fig. S1C–E). First, we performed gait analysis with the CatWalk system to evaluate the stride length and the duty cycle, which represents a ratio of standing duration of each paw in one cycle of walking. In 1–3 weeks after surgery, the stride lengths of the forelimbs and hindlimbs in CSM rats were not different from those in sham-operated rats. In 5–10 weeks after surgery, the stride lengths gradually increased in sham-operated rats, whereas they remained essentially unchanged in CSM rats (Fig. 1C). Similarly, in 5–10 weeks after surgery, the duty cycles gradually decreased in sham-operated rats, whereas they remained stable in CSM rats (Fig. 1D). We also measured the print position, which is the distance between the forepaw step and the hindpaw step. The print positions were substantially unchanged in sham-operated rats, whereas they gradually increased in 5–10 weeks after surgery in CSM rats (Supplementary Fig. 1E). To summarize, CSM rats required a higher number of steps to finish the walkway and kept their paws on the floor for a longer time in walking compared to sham-operated rats.

At 5 weeks after surgery, the number of Nissl body-positive cells in the AH region at C5-6 in CSM rats was lower than that in sham-operated rats at 5 weeks after surgery (Fig. 1B,E). Twy/twy mice develop calcified ligamentum flavum at C2-3, which chronically compresses the cervical cord. The compressed cervical cord shows apoptotic neurons and oligodendrocytes ^15^. Oxidative stress is one of the inducers of apoptosis in CSM^16,34^. We therefore evaluated cellular oxidation by staining for 4-HNE, a marker of lipid peroxidation. The number of 4-HNE-positive neurons was higher at 10 weeks after the surgery in CSM rats compared to that in sham-operated rats (Fig. 1F). As has been reported in previous CSM animal models^28,38^, our CSM model also demonstrated the presence of oxidative stress at the compressed site.

### ZNS ameliorates gait dysfunctions in CSM rat

Zonisamide (ZNS) has a protective effect on the dopaminergic and peripheral neurons against cell death^20,22,25^, and improves motor symptoms in patients with Parkinson's disease^39^. We previously reported in mice that ZNS enhanced neurite elongation of primary spinal motor neurons, and facilitated regeneration of an autograft of the sciatic nerve^20,21^. We thus examined the effects of ZNS on our CSM rats. We intragastrically administered 30 mg/kg/day of ZNS starting at 5 weeks after surgery, when CSM symptoms became overt (Fig. 2A). Daily administration of ZNS for 5 weeks from 5 to 10 weeks after surgery increased the stride lengths in both forelimbs and hindlimbs (Fig. 2B,C). ZNS also decreased the duty cycle of hindlimbs (Fig. 2E). Additionally, the wire mesh walking test showed that daily ZNS administration for 5 weeks suppressed the rate of limb slippage (Fig. 2F and Supplementary Fig. S2). These results demonstrate that daily ZNS administration for 5 weeks improved motor dysfunctions caused by CSM operation.

### ZNS has a neuroprotective effect at the compressed cervical spinal cord

To measure the number of motor neurons, we performed HE staining (Fig. 3A), LFB/Nissl staining (Fig. 3B), and immunohistochemistry with an anti-ChAT antibody (Fig. 4A). The number of cells with Nissl bodies in the AH region in CSM rats without ZNS [ZNS(−)] was lower than that that in sham-operated rats at 10 weeks after surgery (Fig. 3C). In contrast, ZNS administration [ZNS( +)] spared the number of cells with Nissl bodies compared to that in ZNS(−) rats. These results suggest that ZNS reduced the loss of motor neurons in the AH region of CSM rats.

In CSM, myelopathy is caused by demyelination of the pyramidal tracts^40^^,^ as well as the loss of motor neurons. We thus examined the size of the pyramidal tracts in the CSM rats, which are located in the dorsal spinal cord in rodents^41^. We blindly measured the pyramidal tract area by HE staining, and the myelinated area by LFB staining, and found that CSM operation decreased both the pyramidal tract area (Fig. 3D) and the ratio of LFB-positive myelinated area in the pyramidal tract area (Fig. 3E). ZNS administration had no effect on the pyramidal tract area, which was likely because ZNS could not mitigate the compression (Fig. 3D). In contrast, ZNS administration spared the ratio of LFB-positive area in the pyramidal tract area (Fig. 3E). Next, we observed that ZNS administration increased the number of ChAT-positive motor neurons in the spinal cord (Fig. 4A,B). These results suggest that ZNS improved the survival of spinal motor neurons and spared the myelination of motor axons in the spinal cord in CSM rats.

### ZNS increases expressions of anti-oxidants in the spinal cord of CSM rat

We previously reported that ZNS rescued cell death of primary motor neurons exposed to oxidative stress by H_2_O_2_ treatment^20,22,25^. Proliferating cell nuclear antigen (PCNA) is a hallmark of proliferation of reactive astrocytes^42^. A previous report shows that ZNS increases the PCNA expression in 6-hydroxydopamine-treated substantia nigra modeling for Parkinson’s disease^25^. Here we showed that H_2_O_2_ reduced PCNA expression in primary astrocyte derived from normal spinal cord, and ZNS rescued the reduction (Supplementary Fig. S3A). ZNS is thus protective against the oxidative stress in astrocytes, as we previously observed in spinal motor neurons^20,22,25^.

Astrocytes take up cystine via xCT to generate two cysteines, which are then taken up by motor neurons to generate GSH^23,24^. Metallothionein 2A is an anti-oxidant expressed in astrocytes, as well as in motor neurons to a lesser extent^25^. We observed that ZNS increased gene expressions of xCT and metallothionein 2A in primary astrocytes and minimally in the NSC34 motor neuron cell line (Supplemental Figs. S3B-C). Similarly, in the spinal cord of CSM rats at 10 weeks after surgery, ZNS increased gene expressions of xCT and metallothionein 2A (Fig. 5A). We also observed by double immunofluorescence staining for ChAT and GSH that ZNS increased GSH signal intensity in ChAT-positive motor neurons of CSM rats at 10 weeks after surgery (Fig. 5 and Supplemental Fig. 4). Taken together, ZNS induced expressions of anti-oxidants and was likely to have exerted a neuroprotective effect on spinal motor neurons against oxidative stress in CSM rats.

## Discussion

Five different animal models of CSM have been previously reported. First, twy/twy mouse develops ectopic ossification of ligamentum flavum, which compresses the spinal cord. However, the mouse also develops joint ankylosis, which makes it difficult to evaluate motor dysfunctions associated with CSM^15^. Second, axial growth of the cervical spine was restricted by tying a surgical thread around the C4 level. However, this method is technically challenging, and is difficult to induce CSM invariably^43^. Third, a titanium-screw-based device was used to compress the cervical spinal cord. However, elimination of the cervical laminae and step-wise compression of the spinal cord did not recapitulate human CSM^44^. Fourth, synthetic aromatic polyether adsorbs phosphate anions to induce calcium phosphate sedimentation leading to osteoid formation. The degrees of ossification were predicted to be variable from rat to rat, and we had difficulty in obtaining the material^34,38^. Fifth, water-absorbing polyurethane elastomers (Aquaprene C, Aquaprene G, and Aquaprene DX) have been used. We also used Aquaprene DX in our rat model. We hope to note three unique features of our model. First, a 0.7-mm-thick implant has been previously used^9,26,28^. We increased the thickness of the implant to 0.8 mm to enable invariable generation of a rat CSM model. Second, cutting the implant in a blade-shape markedly reduced a chance of developing spinal cord injury. Third, evaluation of motor deficits by the CatWalk system enabled early detection of CSM phenotypes, as has been reported by another group^34,38^. In contrast, evaluation of motor deficits with conventional methods require 8–17 weeks after surgery^26,28^. We confirmed reproducibility of CSM phenotypes in our rat model, and used our model to examine the effect of ZNS.

In our rat CSM model, ZNS rescued motor deficits caused by chronic compression of the spinal cord at C5-6 levels (Fig. 2 and Supplemental Fig. S1). Histologically, ZNS spared the number of spinal motor neurons and ameliorated demyelination of the pyramidal tracts (Figs. 3 and 4). We previously reported that ZNS mitigated H_2_O_2_-induced cell death of primary spinal motor neurons in naive mice^20^. We observed a similar effect of ZNS in primary astrocytes in naive rats (Supplemental Fig. S2A). Amelioration of the development of CSM by ZNS was likely to be accounted for at least partly by induction of expressions of xCT and metallothionein 2A in astrocytes, as well as in neurons to a lesser extent (Supplemental Figs. S2B,C), and also by induction of GSH expression in spinal motor neurons (Figs. 5B,C). ZNS is a pre-approved drug for epilepsy and Parkinson’s disease with accumulated knowledge on optimal doses, adverse effects, and contraindication. The effects of ZNS on motor deficits in patients with CSM need to be scrutinized in accordance with relevant guidelines, but we envisage that ZNS might be able to ameliorate CSM in humans.

## Supplementary information

Supplementary information

## Abbreviations

4-HNE: 4-Hydroxynonenal
AH: Anterior horn
ChAT: Choline acetyltransferase
CNS: Central nervous system
CSM: Cervical spondylotic myelopathy
DAPI: 4,6-Diamidino-2-phenylindole
GSH: Glutathione
PBS: Phosphate buffered saline
PFA: Paraformaldehyde
LFB: Luxol Fast Blue
xCT: Cystine/glutamate exchange transporter
ZNS: Zonisamide

## References

[1] Baptiste DC, Fehlings MG (2006). Pathophysiology of cervical myelopathy. Spine J..

[2] Bohlman HH, Emery SE (1988). The pathophysiology of cervical spondylosis and myelopathy. Spine (Phila Pa 1976).

[3] Wu JC (2013). Epidemiology of cervical spondylotic myelopathy and its risk of causing spinal cord injury: a national cohort study. Neurosurg. Focus.

[4] Karadimas SK, Gatzounis G, Fehlings MG (2015). Pathobiology of cervical spondylotic myelopathy. Eur. Spine J..

[5] Karadimas SK, Erwin WM, Ely CG, Dettori JR, Fehlings MG (2013). Pathophysiology and natural history of cervical spondylotic myelopathy. Spine (Phila Pa 1976).

[6] Nouri A, Tetreault L, Singh A, Karadimas SK, Fehlings MG (2015). Degenerative cervical myelopathy: epidemiology, genetics, and pathogenesis. Spine (Phila Pa 1976).

[7] Klineberg E (2010). Cervical spondylotic myelopathy: a review of the evidence. Orthop. Clin. North Am..

[8] Al-Mefty O (1993). Experimental chronic compressive cervical myelopathy. J. Neurosurg..

[9] Dhillon RS (2016). Axonal plasticity underpins the functional recovery following surgical decompression in a rat model of cervical spondylotic myelopathy. Acta Neuropathol. Commun..

[10] Nagoshi N (2016). Do caucasians and East Asians have different outcomes following surgery for the treatment of degenerative cervical myelopathy? Results from the prospective multicenter AOSpine International Study. Spine (Phila Pa 1976).

[11] Kaminsky SB, Clark CR, Traynelis VC (2004). Operative treatment of cervical spondylotic myelopathy and radiculopathy. A comparison of laminectomy and laminoplasty at five year average follow-up. Iowa Orthop. J..

[12] Kiris T, Kilincer C (2008). Cervical spondylotic myelopathy treated by oblique corpectomy: a prospective study. Neurosurgery.

[13] Papadopoulos CA, Katonis P, Papagelopoulos PJ, Karampekios S, Hadjipavlou AG (2004). Surgical decompression for cervical spondylotic myelopathy: correlation between operative outcomes and MRI of the spinal cord. Orthopedics.

[14] Fehlings MG (2013). Efficacy and safety of surgical decompression in patients with cervical spondylotic myelopathy: results of the AOSpine North America prospective multi-center study. J. Bone Joint Surg. Am..

[15] Yu WR, Liu T, Kiehl TR, Fehlings MG (2011). Human neuropathological and animal model evidence supporting a role for Fas-mediated apoptosis and inflammation in cervical spondylotic myelopathy. Brain.

[16] Fehlings MG, Skaf G (1998). A review of the pathophysiology of cervical spondylotic myelopathy with insights for potential novel mechanisms drawn from traumatic spinal cord injury. Spine (Phila Pa 1976).

[17] Mizuno J, Nakagawa H, Inoue T, Hashizume Y (2003). Clinicopathological study of "snake-eye appearance" in compressive myelopathy of the cervical spinal cord. J. Neurosurg..

[18] Tetreault L (2018). Significant predictors of outcome following surgery for the treatment of degenerative cervical myelopathy: a systematic review of the literature. Neurosurg. Clin. N. Am..

[19] Murata M (2004). Novel therapeutic effects of the anti-convulsant, zonisamide, on Parkinson's disease. Curr. Pharm. Des..

[20] Yagi H (2015). Zonisamide enhances neurite elongation of primary motor neurons and facilitates peripheral nerve regeneration in vitro and in a mouse model. PLoS ONE.

[21] Ohno K, Yagi H, Ohkawara B (2016). Repositioning again of zonisamide for nerve regeneration. Neural Regen. Res..

[22] Asanuma M (2010). Neuroprotective effects of zonisamide target astrocyte. Ann. Neurol..

[23] Wang XF, Cynader MS (2000). Astrocytes provide cysteine to neurons by releasing glutathione. J. Neurochem..

[24] Shih AY (2006). Cystine/glutamate exchange modulates glutathione supply for neuroprotection from oxidative stress and cell proliferation. J. Neurosci..

[25] Choudhury ME (2012). Zonisamide up-regulated the mRNAs encoding astrocytic anti-oxidative and neurotrophic factors. Eur. J. Pharmacol..

[26] Ijima Y (2017). Experimental rat model for cervical compressive myelopathy. NeuroReport.

[27] Yamamoto S, Kurokawa R, Kim P (2014). Cilostazol, a selective Type III phosphodiesterase inhibitor: prevention of cervical myelopathy in a rat chronic compression model. J. Neurosurg. Spine.

[28] Kim P, Haisa T, Kawamoto T, Kirino T, Wakai S (2004). Delayed myelopathy induced by chronic compression in the rat spinal cord. Ann. Neurol..

[29] Bradbury EJ (2002). Chondroitinase ABC promotes functional recovery after spinal cord injury. Nature.

[30] Imagama S (2011). Keratan sulfate restricts neural plasticity after spinal cord injury. J. Neurosci..

[31] Hamers FP, Lankhorst AJ, van Laar TJ, Veldhuis WB, Gispen WH (2001). Automated quantitative gait analysis during overground locomotion in the rat: its application to spinal cord contusion and transection injuries. J. Neurotrauma.

[32] Koopmans GC (2005). The assessment of locomotor function in spinal cord injured rats: the importance of objective analysis of coordination. J. Neurotrauma.

[33] Moon ES, Karadimas SK, Yu W-R, Austin JW, Fehlings MG (2014). Riluzole attenuates neuropathic pain and enhances functional recovery in a rodent model of cervical spondylotic myelopathy. Neurobiol. Dise..

[34] Karadimas SK (2015). Riluzole blocks perioperative ischemia-reperfusion injury and enhances postdecompression outcomes in cervical spondylotic myelopathy. Sci. Transl. Med..

[35] Nagai T (2018). Analysis of spastic gait in cervical myelopathy: Linking compression ratio to spatiotemporal and pedobarographic parameters. Gait Posture.

[36] Maezawa Y, Uchida K, Baba H (2001). Gait analysis of spastic walking in patients with cervical compressive myelopathy. J. Orthop. Sci..

[37] Suzuki E, Nakamura H, Konishi S, Yamano Y (2002). Analysis of the spastic gait caused by cervical compression myelopathy. J. Spinal Disord. Tech..

[38] Karadimas SK (2013). A novel experimental model of cervical spondylotic myelopathy (CSM) to facilitate translational research. Neurobiol. Dis..

[39] Murata M, Horiuchi E, Kanazawa I (2001). Zonisamide has beneficial effects on Parkinson's disease patients. Neurosci. Res..

[40] Ito T, Oyanagi K, Takahashi H, Takahashi HE, Ikuta F (1996). Cervical spondylotic myelopathy. Clinicopathologic study on the progression pattern and thin myelinated fibers of the lesions of seven patients examined during complete autopsy. Spine (Phila Pa 1976).

[41] Terashima T, Ochiishi T, Yamauchi T (1994). Immunohistochemical detection of calcium/calmodulin-dependent protein kinase II in the spinal cord of the rat and monkey with special reference to the corticospinal tract. J. Comp. Neurol..

[42] Miyake T, Okada M, Kitamura T (1992). Reactive proliferation of astrocytes studied by immunohistochemistry for proliferating cell nuclear antigen. Brain Res..

[43] Kubota M (2011). Development of a chronic cervical cord compression model in rat: changes in the neurological behaviors and radiological and pathological findings. J. Neurotrauma.

[44] Lee J, Satkunendrarajah K, Fehlings MG (2012). Development and characterization of a novel rat model of cervical spondylotic myelopathy: the impact of chronic cord compression on clinical, neuroanatomical, and neurophysiological outcomes. J. Neurotrauma.

