# Supplementary material for: Zonisamide ameliorates progression of cervical spondylotic myelopathy in a rat model
Source: Sci Rep. 2020 Aug 4;10:13138. doi: 10.1038/s41598-020-70068-0 (PMC7403578; doi:10.1038/s41598-020-70068-0)

Zonisamide ameliorates progression of cervical spondylotic myelopathy in a rat model

Shunsuke Kanbara[1,2], Bisei Ohkawara[2,#], Hiroaki Nakashima[1], Kyotaro Ota[1,2], Hiroyuki Koshimizu[1,2], Taro Inoue[1,2], Hiroyuki Tomita[1,2], Mikako Ito[2], Akio Masuda[2], Naoki Ishiguro[1], Shiro Imagama[1], Kinji Ohno[2]

[1]Department of Orthopedic Surgery, Nagoya University Graduate School of Medicine, Nagoya, Japan, 65 Tsurumai, Showa-ku, Nagoya 466-8550, Japan
Phone: +81-52-744-1908, Fax: +81-52-744-2785
[2]Division of Neurogenetics, Center for Neurological Diseases and Cancer, Nagoya University Graduate School of Medicine, Nagoya, Japan, 65 Tsurumai, Showa-ku, Nagoya 466-8550, Japan
Phone: +81-52-744-2447, Fax: +81-52-744-2449

[#]Corresponding author
Bisei Ohkawara, PhD, e-mail: biseiohkawara@med.nagoya-u.ac.jp

E-mail list for authors:
Shunsuke Kanbara, MD, e-mail: kanbarashunsuke@med.nagoya-u.ac.jp
Kyotaro Ota, MD, e-mail: okyotaro@med.nagoya-u.ac.jp
Hiroaki Nakashima, MD, PhD, e-mail: hirospine@gmail.com
Hiroyuki Koshimizu, MD, e-mail: hkoshimizu@med.nagoya-u.ac.jp
Taro Inoue, MD, e-mail: inoue.taro@h.mbox.nagoya-u.ac.jp
Hiroyuki Tomita, MD, e-mail: tomita.hiroyuki@k.mbox.nagoya-u.ac.jp
Mikako Ito, PhD, e-mail: ito@med.nagoya-u.ac.jp
Akio Masuda, MD, PhD, e-mail: amasuda@med.nagoya-u.ac.jp
Naoki Ishiguro, MD, PhD, e-mail: n-ishi@med.nagoya-u.ac.jp
Shiro Imagama, MD, PhD, e-mail: imagama@med.nagoya-u.ac.jp
Kinji Ohno, MD, PhD, e-mail: ohnok@med.nagoya-u.ac.jp

Supplementary Information

Materials and Methods

Isolation of primary astrocytes

Primary cultures of spinal astrocytes were prepared as described previously [1]. Five female or male pups (0- to 2-day-old) of Wistar rats were immediately decapitated and the isolated spinal cord was minced in divalent cation-free Hanks' balanced salt solution (Nacalai). The minced spinal cords were harvested by grinding on a cell strainer (40 μm) (Falcon, 352340) with instillation of cation-free Hanks' balanced salt solution [HBSS(-), Nacalai tesque, 1746-15]. The cells were centrifuged for 5 min at 270 x *g* to remove debris in the supernatant. The cells were suspended with HBSS(-) including papain (10 U/ml, WAKO, 164-00172), and were left at room temperature for 10 min. Then, the solution was added with DNase (0.1 mg/ml, Sigma, DN25), and was left at room temperature for 10 min. After the enzymatic dissociation, the tissues were mechanically dissociated with a Pasteur pipette in culture medium. Samples were centrifuged for 5 min at 270 x *g*, and the culture medium was added after dumping the supernatants. Cells were cultured in Dulbecco’s modified Eagle’s medium (DMEM)/F-12 (Gibco) containing 10% fetal bovine serum (FBS, Sigma-Aldrich, 172012) and 1% of Pen Strep (10,000 U/ml, Gibco, 151400-122). Cells were seeded and maintained for 2 weeks in culture medium in a T-75 poly-L-lysine-coated culture flask (Sigma-Aldrich) at 37°C in 5% CO_2. Culture medium was changed three times a week. When cells reached 80-90% confluency, the flask was shaken vigorously at 250 rpm for at least 12 h to remove non-astrocytic cells. The remaining astrocytes were seeded into a new poly-L-lysine-coated plate.

Culture of NSC34 cells

NSC34 cells (mouse neuroblastoma-spinal cord hybrid cells displaying a multipolar motor neuron-like phenotype) that stably expressed the doxycycline-induced green fluorescent protein (GFP) construct (NSC34-pTetR12-TO / GFP) [2] were kindly provided by Dr. Shinsuke Ishigaki, Department of Neurology, Nagoya University Graduate School of Medicine, and were cultured as previously described [3]. Briefly, NSC34 cells were cultured in a humidified atmosphere with 95% air-5% CO_2 in a 37°C incubator in DMEM supplemented with 10% FBS (Sigma-Aldrich, 172012) for proliferation.

References

1. Eguchi, R., et al., Different mechanisms of extracellular adenosine accumulation by reduction of the external Ca(2+) concentration and inhibition of adenosine metabolism in spinal astrocytes. J Pharmacol Sci, 2015. **128**(1): p. 47-53.
2. Asanuma, M., et al., Neuroprotective effects of zonisamide target astrocyte. Ann Neurol, 2010. **67**(2): p. 239-49.
3. Wang, X.F. and M.S. Cynader, Astrocytes provide cysteine to neurons by releasing glutathione. J Neurochem, 2000. **74**(4): p. 1434-42.

Supplemental Table S1. Primer sequences for qRT-PCR

Rat gene	Primer sequence
Slc7a11 (xCT)	For. 5' CATTGTATGGGACAAGAAACC 3'
	Rev. 5' GGCACTAGACTCAAGAACTGT 3'
Mt2a (metallothionein 2A)	For. 5' CACAGATGGATCCTGCTCCT 3'
	Rev. 5' GAGAACCGGTCAGGGTTGTA 3'
Pcna (PCNA)	For. 5' TAAGTTGTCCCAGACAAGCA 3'
	Rev. 5' GCGATCGTCAAAGGTTTAGT 3'
Actb (β actin)	For. 5' TCTACAATGAGCTGCGTGTG 3'
	Rev. 5' TACATGGCTGGGGTGTTGAA 3'

For., forward primer; Rev., reverse primer

Supplemental Figure legends

Supplemental Figure S1. Water-absorbing polyurethane blade for CSM operation in rats

(A) Volumetric measurement of expansion of a blade-shaped water-absorbing polyurethane elastomer (Aquaprene Dx) implanted under the rat skin. The blade gradually expanded and reached a volume of 150% in 24 h. Mean and SD are indicated ($n = 3$). **(B)** Frontal/lateral views and schematic presentation of the blade before implantation. A slanted edge was made at the corner not to damage the spinal cord during implantation. A thread was added to the blade to smoothly place the blade underneath the C5 and C6 laminae. **(C)** Experimental protocol of gait and histological analyses of both sham-operated and CSM rats. Water-absorbing polyurethane blade was implanted in CSM rats at 12 weeks of age. **(D)** Dorsal view of a representative intraoperative image. Following the resection of ligamentum flavum at C5–6 and C6–7, the blade passed through the resected C6-7 space and epidurally slipped underneath the C5 and C6 laminae without laminectomy. **(E)** Gait analysis with CatWalk to measure the distance (“print position”) between the forepaw step and the hindpaw step (yellow double-headed arrow in a representative CatWalk image). Print positions extended from 5 weeks after surgery in CSM rats. Mean and SE are indicated ($n = 6$ rats in each group).

Supplemental Figure S2. Effects of Zonisamide (ZNS) on gene expressions of *Pcna* encoding proliferating cell nuclear antigen (PCNA), *Slc7a11* encoding cystine/glutamate exchange transporter (xCT), and *Mt2a* encoding metallothionein

2A in primary astrocytes and in NSC34 neuronal cells.

Primary astrocytes isolated from the rat spinal cord around C5-6 on postnatal day 1 were cultured with growth media. NSC34 cells were cultured with differentiation medium for 3 days. Primary astrocytes and NSC34 cells were treated with 10 µM ZNS. After 1 h, cells were added with 100 µM H_2O_2 for 24 h. Gene expressions of *Pcna* encoding proliferating cell nuclear antigen **(A)**, *Slc7a11* encoding cystine/glutamate exchange transporter (xCT, **B**), and *Mt2a* encoding metallothionein 2A **(C)** were normalized for the expression of *Actb* encoding β-actin. Mean and SD are indicated (n = 3 culture dishes in each group). *$p < 0.05$ and **$p < 0.01$ by Kruskal-Wallis test followed by Dunn-Bonferroni HSD.

Supplemental Figure S3. Effects of Zonisamide (ZNS) on immunostaining for glutathione (GSH) in ChAT-positive spinal motor neurons in CSM rats.

Low- and high-magnification images for Fig. 5B of GSH (green)- and ChAT (red)-positive cells in the anterior horn (AH) region of the spinal cord at the C5-6 disc level in CSM rats with [ZNS (+), B] or without [ZNS (-), A] ZNS administration. White arrowhead indicate ChAT-positive motor neurons in the AH region. Bars = 50 µm

Supplemental Figure 1

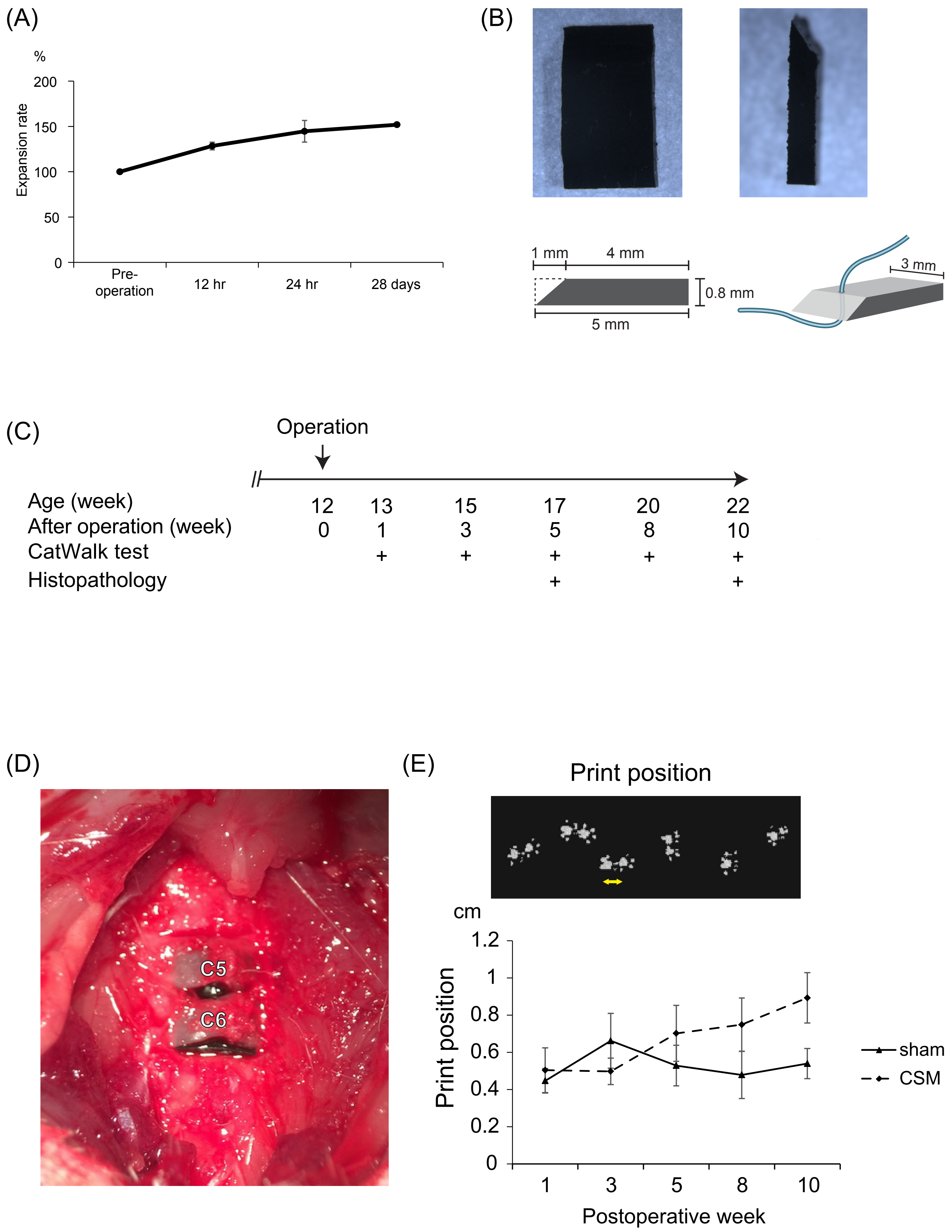

Supplemental Figure 2

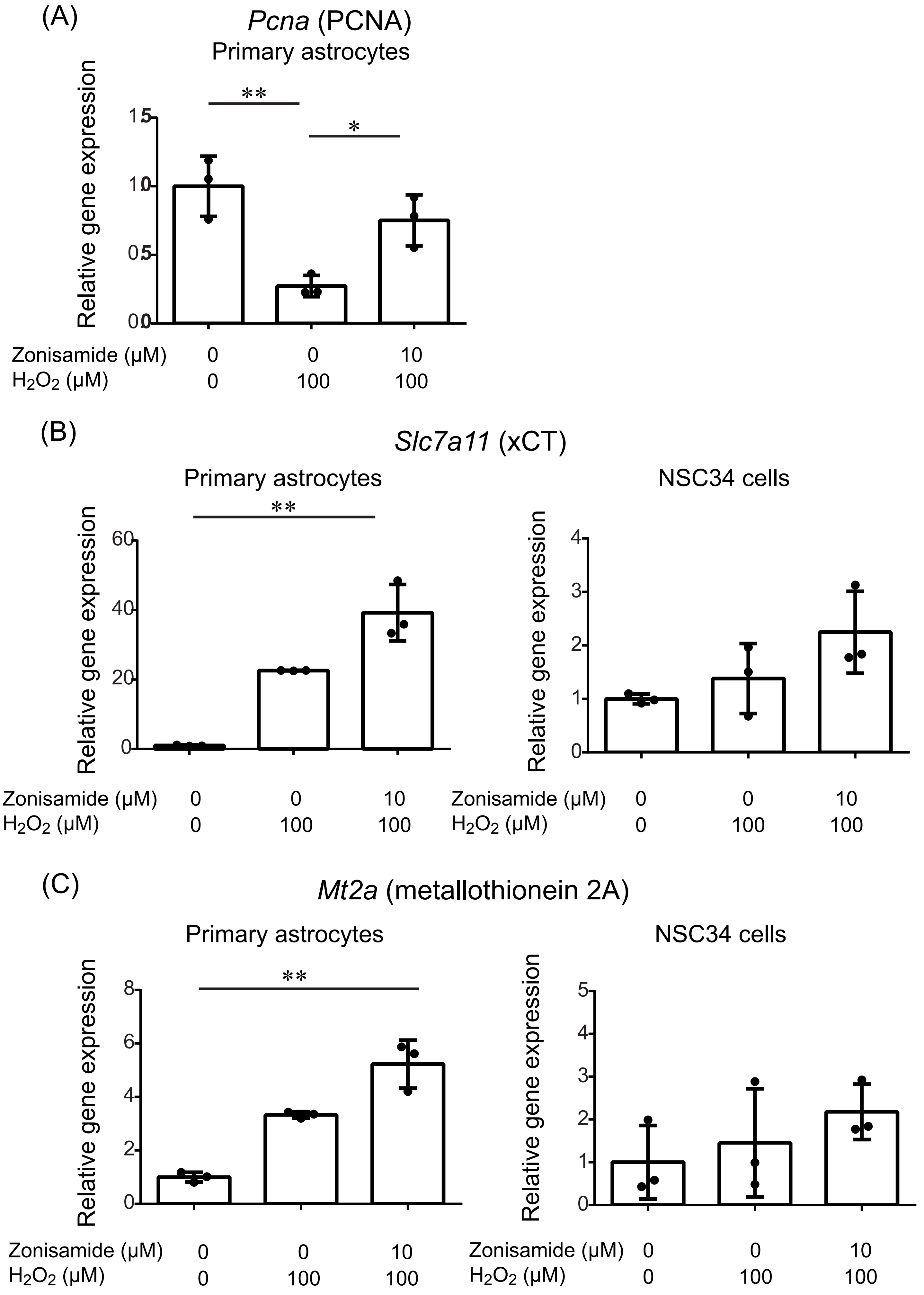

Supplemetal Figure 3

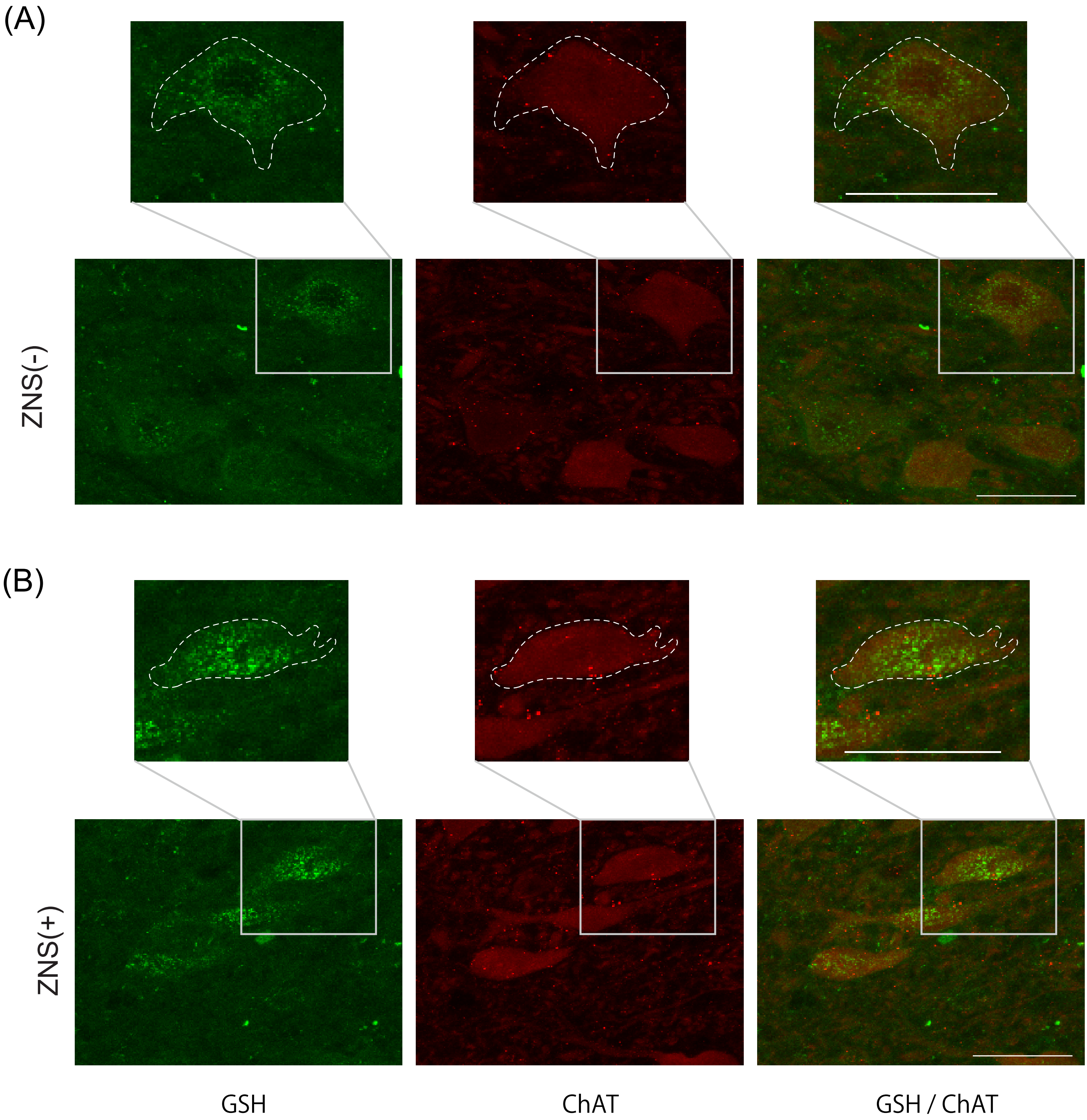

Supplement: Supplementary file 1 — Supplementary information [file 41598_2020_70068_MOESM1_ESM.pdf]
